# Questing ixodid ticks on the vegetation of sable antelope and multi-herbivore enclosures in Thabazimbi

**DOI:** 10.4102/jsava.v86i1.1243

**Published:** 2015-07-14

**Authors:** Andre C. Uys, Ivan G. Horak, Alan Harrison

**Affiliations:** 1 The Marakele Park (PTY) Ltd., Thabazimbi, South Africa; 2 Department of Veterinary Tropical Diseases, University of Pretoria, South Africa; 3 Institute of Biological and Environmental Sciences, Department of Zoology, University of Aberdeen, United Kingdom

## Abstract

This survey of ixodid ticks was the first to compare the species composition and population dynamics of free-living ticks in intensive, sable antelope breeding enclosures, now commonplace in commercial wildlife ranching in South Africa, with those of multi-herbivore enclosures. The species composition, abundance and seasonal abundance of questing ixodid ticks on the vegetation in intensive breeding enclosures for sable antelope (*Hippotragus niger*), on which strategic tick control is practised, were compared with those of ticks in a multi-species herbivore enclosure surrounding the breeding enclosures in which no tick control is practised. A total of eight ixodid tick species were collected by drag-sampling the woodland and grassland habitats in each enclosure type monthly from July 2011 to July 2013. *Rhipicephalus decoloratus,* a potential vector of fatal tick-borne disease in sable antelopes, was the most abundant, accounting for 65.4% of the total number of ticks collected in the sable enclosures, whilst representing only 25.4% of number of ticks collected in the multi-species herbivore enclosure. *Rhipicephalus decoloratus* and *R. evertsi evertsi* were more abundant than *R. appendiculatus* (both *p* < 0.05) and *Amblyomma hebraeum* (*p* < 0.001 and *p* < 0.01, respectively). *Rhipicephalus decoloratus* larvae were collected throughout the year, with peak collections in November 2012 and October to December 2013 in the sable enclosures; and in April/May 2012 and February/April 2013 in the multi-species herbivore enclosure. More *R. decoloratus* were recovered in the second year than in the first year in the grassland habitat of the sable enclosures (*V* = 7.0, *p* < 0.05) possibly as a result of acaricide resistance. The apparent temporal over-abundance of *R. decoloratus* in sable antelope breeding enclosures, in the face of strategic tick control, is of concern and requires further investigation.

## Introduction

### Problem statement

Commercial wildlife ranching has become increasingly popular in the past two decades and has, to a large extent, displaced commercial cattle farming in the bushveld regions of Limpopo Province, South Africa (Schroder, Uys & Reilly 2006). Moreover, commercial game ranching in this country has adopted the practice of confining rare and endangered species such as sable antelope (*Hippotragus niger*) and roan antelope (*Hippotragus equinus*) in intensive breeding enclosures in order to stimulate reproduction and minimise neonatal mortality, which, because of tick-borne diseases, is high in these species (Nijhof *et al.* 2005). Neonatal and calf mortalities have, however, not been eliminated by the confinement and intensification of breeding of these species and have, in some cases, even been exacerbated.

Roan and sable antelope have a low tolerance to competition from other herbivore species and are usually accommodated in mono-species intensive breeding enclosures, varying in size from 10 ha to 150 ha. Breeding enclosures are fenced to exclude predators and other herbivorous species. In addition, animals in the enclosures are subject to tick control and are maintained through dry periods by supplementary feeding (Schroder *et al.* 2006). Commercial ranching of these species has resulted in an increase in the numbers of animals translocated within South Africa and this, in turn, has led to the introduction of hosts and/or ticks into non-endemic areas, resulting in mortalities from tick-borne diseases, tick toxicoses, tick worry and anaemia (McInnes *et al.* 1991;, Nijhof *et al.* 2005). Mortalities as a result of tick-borne disease have been reported in a number of wildlife species, including sable and roan antelope, but little is known about which tick species transmit these diseases (Grootenhuis *et al.* 1980;, Nijhof *et al.* 2003, 2005;, Oosthuizen *et al.* 2008).

*Rhipicephalus appendiculatus, Rhipicephalus decoloratus* and *Rhipicephalus evertsi evertsi* were the most common tick species collected from confirmed cases of fatal theileriosis in roan and sable antelope (Wilson & Hirst 1977). *Rhipicephalus appendiculatus* and *R. evertsi evertsi* have recently been proposed as potential vectors of *Theileria* spp. (sable), the causative organism of mortalities in both roan and sable antelope, whilst the role of *R. decoloratus* in its transmission is unclear (Benade 2010;, Steyl *et al.* 2012). Moreover, *R. decoloratus* and *R. evertsi evertsi* were the only tick species recovered from sable antelope that died during an outbreak of clinical babesiosis in an intensive breeding enclosure in which the animals were under nutritional stress (A.C. Uys, unpublished data 2006). Determining the epidemiology of important tick-borne diseases in wildlife and identifying the tick vectors responsible for the transmission of these diseases will require reliable data on the preferred host status and seasonality of the potential tick vectors, as well as their distribution in relation to disease outbreaks (Oosthuizen *et al.* 2009).

#### Trends

A non-destructive, non-invasive method of determining the species composition and seasonality of tick populations questing for hosts is by drag-sampling the vegetation with flannel strips. Ticks differ in their host-finding strategies, thus not all tick species, or even all stages of a particular species, can be collected by drag-sampling. For instance, only the larvae of *Amblyomma hebraeum*, a three-host tick, quest for hosts from the vegetation, whereas the nymphs and adults actively hunt for hosts from the soil surface or leaf litter. The larvae, nymphs and adults of the three-host tick, *R. appendiculatus*, all quest for hosts from the vegetation, whilst only the larvae of the one-host *R. decoloratus* quest from the vegetation. Larvae of the two-host tick, *R. evertsi evertsi,* quest for hosts from the vegetation and the adults probably from the soil surface (Gallivan *et al.* 2011; Horak, Gallivan & Spickett 2011; Spickett, Gallivan & Horak 2011).

Drag-sampling has been carried out in numerous wildlife reserves in southern Africa as well as on commercial game ranches (Schroder *et al.* 2006; Spickett *et al.* 1991, 1992; Uys & Horak 2005; Zieger, Horak & Cauldwell 1998a). No such surveys have been performed comparing free-living ixodid tick populations in intensive mono-species wildlife breeding systems with those found on surrounding multi-herbivore species ranches.

The population dynamics of ticks are influenced by complex relationships and interactions between them, their hosts and the environment (Spickett *et al.* 2011). Long-term surveys of questing free-living ixodid ticks in the Kruger National Park have detected significant erratic and periodic declines and increases in their numbers in response to climatic factors, host density, host species composition and host resistance (Horak *et al.* 2011). Drag-sampling of the vegetation over a 4-year period on a commercial game ranch in Limpopo Province, on which intensive breeding and strategic tick control is practised, has revealed changes in the species composition of free-living ixodid ticks in single-species breeding camps. Regular acaricide application on cattle on a mixed wildlife and cattle ranch in Zambia reduced populations of parasitic ticks on sympatric impalas (*Aepyceros melampus*) and free-living ticks on the vegetation (Zieger *et al.* 1998c). However, *R. decoloratus* appeared to be unaffected, possibly because of acaricidal resistance (Zieger *et al.* 1998c).

#### Objectives

A comparison between the questing tick populations in intensive breeding enclosures for sable antelope – on which tick control is practised – and similar tick populations on a commercial game ranch with multiple herbivore species surrounding the sable enclosures, should assist in determining the species composition and the population dynamics of ticks considered to be potential vectors of fatal tick-borne diseases in these animals.

## Research method and design

### Setting

The survey was conducted on a commercial game ranch, Hoopdal KQ96 (S24°17.993 E027°29.365), Thabazimbi district, Limpopo Province. The vegetation on the ranch is classified as Western Sandy Bushveld of the Savanna Biome with erratic rainfall varying between 450 mm and 750 mm per annum (Mucina & Rutherford 2006). The farm comprises approximately 1900 ha and is subdivided into an enclosure of approximately 1070 ha in which several herbivore species are contained, as well as three sable antelope breeding enclosures of approximately 50 ha each. One adult male and 10–15 adult female sable antelope and their offspring are kept in each of the three breeding enclosures. Regular tick control is practised in these enclosures by treating the sables every 2–3 weeks with a synthetic pyrethroid applied by means of a specially-designed square feeding trough that is framed with a gutter into which the acaricide is poured. The acaricide-filled gutter contains a steel rolling pin, which becomes coated in acaricide and rubs against the animal’s neck when it leans over the pin to feed.

Wildlife species in the multiple herbivore area include: 63 plains zebra (*Equus quagga*), unknown numbers of warthogs (*Phacochoerus africanus*), 15 giraffe (*Giraffa camelopardalis*), 46 greater kudu (*Tragelaphus strepsiceros*), 5 eland (*Tragelaphus oryx*), 5 blue wildebeest (*Connochaetes taurinus*), 12 gemsbok (*Oryx gazella*), 27 waterbuck (*Kobus ellypsiprimnus*), an unknown number of common duiker (*Sylvicapra grimmia*), an unknown number of steenbok (*Raphicerus campestris*) and 93 impala (*Aepyceros melampus*). The African buffalo (*Syncerus caffer*) that were present on the farm prior to 2008 have all been removed and the property has been buffalo-free since then. Moreover, the intensive breeding enclosures have also stood empty since 2008 and were only restocked in 2011. In the same year, the numbers of plains wildlife were reduced when 13 zebra, 11 kudu, 46 eland, 10 waterbuck and 53 impala were removed for live sale. No tick control is practised in the multi-species herbivore enclosure.

### Procedure

#### Drag-sampling

With the exception of January 2013, when no collections were made, drag-sampling of the vegetation in the sable antelope breeding enclosures and the multiple herbivore species enclosure was carried out in the third week of each month, starting in July 2011 and continuing until July 2013. Drag-sampling, which favours the collection of questing ixodid tick larvae, was accomplished by dragging flannel strips over the vegetation (Petney & Horak 1987; Spickett *et al.* 1991). Each drag-sampling event included 3x250 m-long drags of the woodland and three of the grassland habitats in the multiple herbivore species area, as well as a single drag in the woodland and the grassland in each of the three intensive sable antelope breeding enclosures. Sharp-pointed forceps were used to remove the ticks from the flannel strips after each drag, after which they were stored in 70% ethanol in internally-labelled glass screw-top vials for later identification and counting.

#### Tick identification

The ticks that had been collected were identified and counted in a Perspex tray with a grid pattern on its base under an Olympus VMZ 1–4x stereoscopic dissecting microscope. The larvae of *A. hebraeum*, *A. marmoreum*, *R. decoloratus*, *R. evertsi evertsi* and *R. zambeziensis*, as well as the larvae and nymphs of *R. appendiculatus* and *R. simus* and the adults of *Haemaphysalis elliptica*, were identified by means of the descriptions published by various authors (Apanaskevich, Horak & Camicas 2007; Arthur 1973, 1975; Gothe 1967; Voltzit & Keirans 2003; Walker, Keirans & Horak 2000). Moreover, the senior and last authors have several years of personal experience in identifying ticks collected by drag-sampling.

### Analyses

#### Statistical methods

With a few exceptions, only larvae were collected from the vegetation, hence the statistical comparisons of the relative abundance of tick species took only larvae into account. In order to examine the effects of habitat type, the total number of ticks collected in woodland transects were compared with that of grassland transects for each individual tick species within each enclosure type using a Wilcoxon signed rank test paired by the month of collection. The same statistical approach was used to examine the effect of enclosure type where the total number of ticks collected in multi-species enclosures was compared with that of mono-species enclosures for both woodland and grassland habitats for each tick species.

A Friedman test with cases paired by month was used to determine if there were any overall differences in the relative abundance of tick species in each habitat/enclosure combination (woodland/multi-species, grassland/multi-species, woodland/mono-species, grassland/mono-species). Friedman tests that gave significant results were examined further with pairwise Wilcoxon signed rank tests, again paired by month, but with Bonferroni correction for multiple comparisons, thus identifying where significant differences occurred between tick species. Data yielding significant differences between groups are presented in boxplots.

To establish whether any temporal differences presented in the abundance of ticks, the total abundance of each tick species between the first (July 2011–June 2012) and second year (July 2012–July 2013) of the study was compared with the others using Wilcoxon signed rank tests paired by month for each habitat/enclosure type combination. As ticks were not sampled in all months of each year, the months January 2012 and July 2013 were removed from this analysis to permit the use of a paired design.

## Results

### Total ticks

**FIGURE 1:**
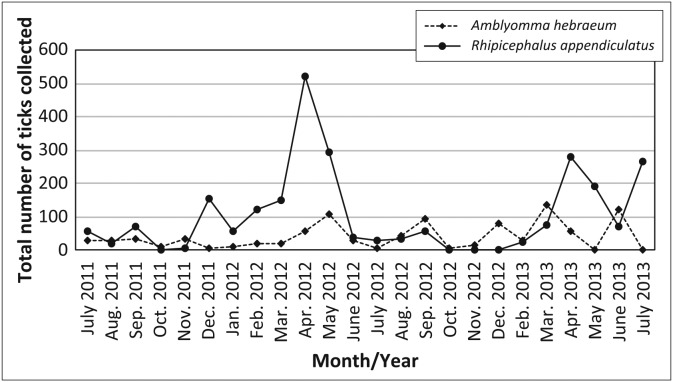
Total numbers of *Amblyomma hebraeum* and *Rhipicephalus appendiculatus* larvae collected between July 2011 and July 2013 by monthly drag-sampling of the vegetation on the farm Hoopdal KQ96.

**FIGURE 2:**
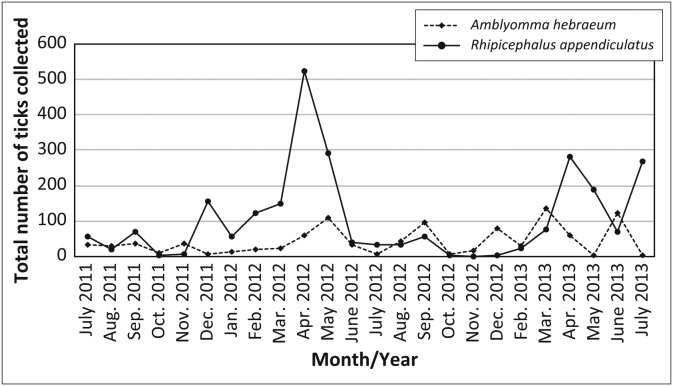
Total numbers of *Rhipicephalus decoloratus* and *Rhipicephalus evertsi evertsi* larvae collected between July 2011 and July 2013 by monthly drag-sampling of the vegetation on the farm Hoopdal KQ96 between July 2011 and July 2013.

During the 24-month sampling period, a total of 10 399 ticks were collected from the vegetation of the woodland and grassland habitats in the multi-species herbivore enclosure and the sable antelope breeding enclosures (Table 1). More ticks (*n* = 6610; 63.6%) were collected in the multi-species herbivore enclosure than in the sable antelope enclosures (*n* = 3489; 33.5%); and more ticks (*n* = 5896; 56.7%) were collected in the grasslands than in the woodlands (*n* = 4503; 43.3%). *Rhipicephalus decoloratus* was the most commonly collected tick and accounted for 38.1% (*n* = 3957) of all ticks collected, followed by *R. evertsi evertsi* (*n* = 2866; 27.6%), *R. appendiculatus* (*n* = 2515; 24.2%) and *A. hebraeum* (*n* = 989; 9.5%). *Rhipicephalus decoloratus*, which was collected throughout the year, was the most abundant tick in the intensive sable antelope breeding enclosures, accounting for 65.4% (*n* = 2281) of all ticks collected in these enclosures. *Rhipicephalus appendiculatus* was the most abundant tick collected in the multi-species herbivore enclosure (*n* = 2291; 34.7%), followed by *R. evertsi evertsi* (*n* = 2047; 31.0%) and *R. decoloratus* (*n* = 1676; 25.4%). The total numbers of *A. hebraeum* and *R. appendiculatus* larvae collected are depicted in Figure 1 and the total numbers of *R. decoloratus* and *R. evertsi evertsi* larvae collected are depicted in Figure 2.

**TABLE 1:** Total number of ticks collected from the woodland and grassland habitats in the multi-species herbivore enclosure and sable antelope breeding enclosures on the farm Hoopdal KQ96 between July 2011 and July 2013.

Tick species	Number of ticks collected in multi-species herbivore enclosure		Number of ticks collected in sable antelope enclosures		Total
	Woodland	Grassland	Woodland	Grassland	
*Amblyomma hebraeum*	430	432	104	23	989
*Amblyomma marmoreum*	0	0	2	0	2
*Haemaphysalis elliptica*	2	0	0	0	2
*Rhipicephalus appendiculatus*	1489	802	122	102	2515
*Rhipicephalus decoloratus*	411	1265	1044	1237	3957
*Rhipicephalus evertsi evertsi*	413	1634	434	385	2866
*Rhipicephalus simus*	2	0	3	2	7
*Rhipicephalus zambeziensis*	21	9	26	5	61
**Subtotals**	**2768**	**4142**	**1735**	**1754**	**-**
**Totals**	**6910**		**3489**		**10 399**

**FIGURE 3:**
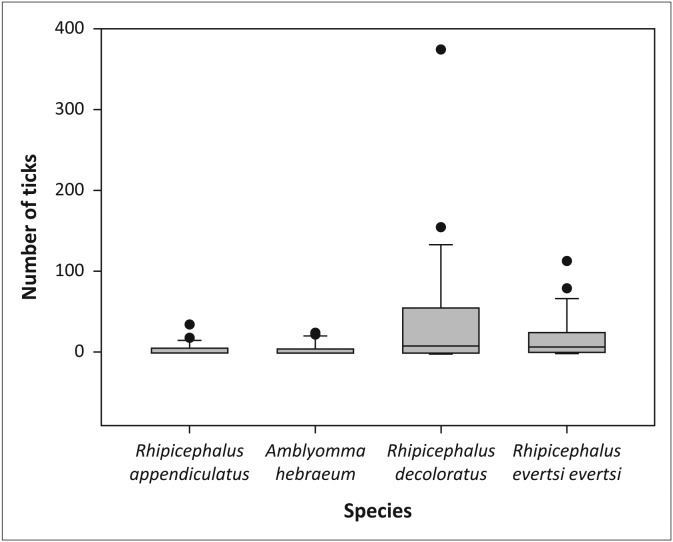
Boxplot depicting the significant difference in abundance between individual tick species in the woodland of the sable antelope breeding enclosures on the Farm Hoopdal KQ96. The middle bar, the box and the whiskers refer to the median, the interquartile range and the interoctile (80%) range, whilst the dots are extremes or outliers.

**FIGURE 4:**
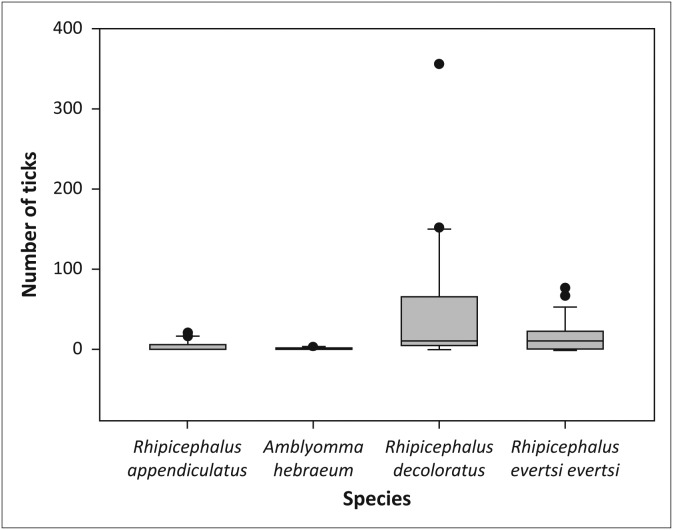
Boxplot depicting the significant difference in abundance between individual tick species in the grassland of the sable antelope breeding enclosures on the Farm Hoopdal KQ96. The middle bar, the box and the whiskers refer to the median, the interquartile range and the interoctile (80%) range, whilst the dots are extremes or outliers.

There was a significant overall difference in the abundance of individual tick species in the woodland habitat in the sable enclosures (*χ*^2^ = 13.42, *df* = 3, *p* < 0.01) and post-hoc analysis (pairwise Wilcoxon signed rank tests with Bonferroni correction) demonstrated that *R. decoloratus* was more abundant than both *R. appendiculatus* (*p* < 0.05) and *A. hebraeum* (*p* < 0.05) (Figure 3). There was also a difference in the overall abundance of tick species in the grassland in the sable enclosures (*χ*^2^ = 23.06, *df* = 3, *p* < 0.001). Post-hoc analysis showed that *R. decoloratus* and *R. evertsi evertsi* were more abundant than *R. appendiculatus* (both *p* < 0.05) and *A. hebraeum* (*p* < 0.001 and *p* < 0.01, respectively) (Figure 4).

#### Amblyomma hebraeum

**FIGURE 5:**
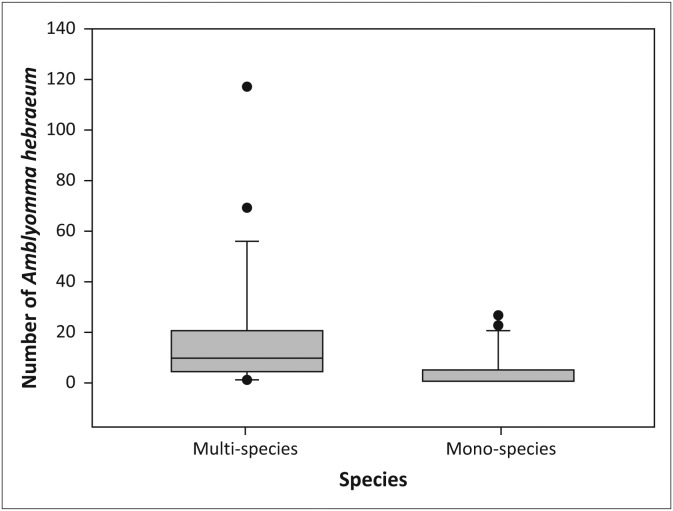
Boxplot depicting the number of *Amblyomma hebraeum* larvae collected from the vegetation in the woodland in the multi-species enclosure versus the sable antelope enclosures on the farm Hoopdal KQ96. The middle bar, the box and the whiskers refer to the median, the interquartile range and the interoctile (80%) range, whilst the dots are extremes or outliers.

**FIGURE 6:**
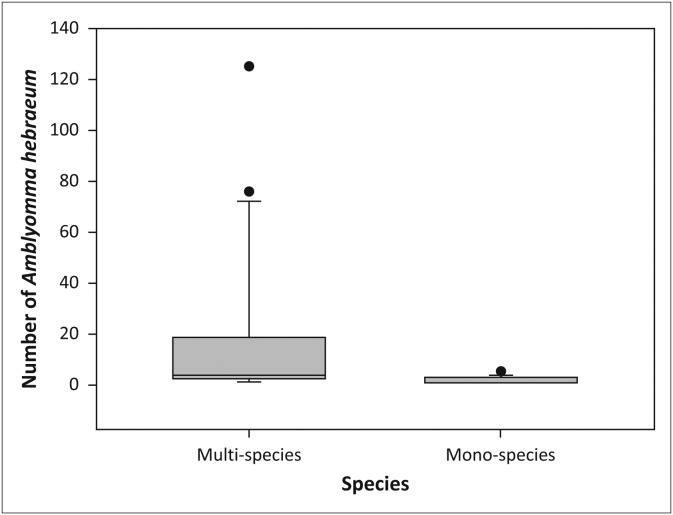
Boxplot depicting the number of *Amblyomma hebraeum* larvae collected from the vegetation in the grassland in the multi-species enclosure versus the sable antelope enclosures on the farm Hoopdal KQ96. The middle bar, the box and the whiskers refer to the median, the interquartile range and the interoctile (80%) range, whilst the dots are extremes or outliers.

**FIGURE 7:**
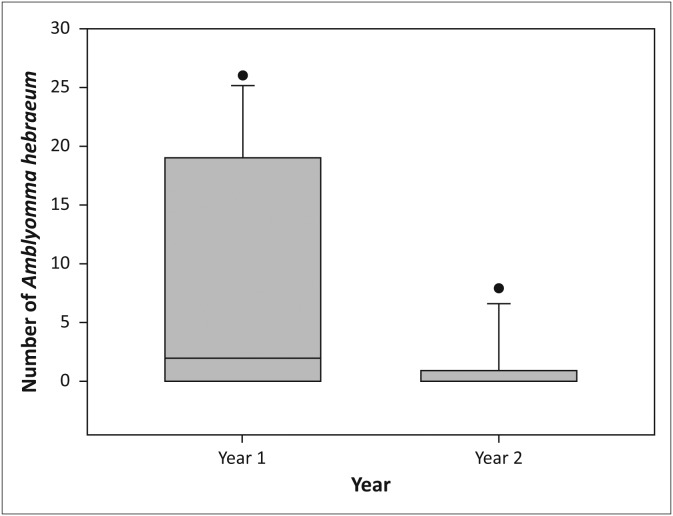
Boxplot depicting higher numbers of *Amblyomma hebraeum* larvae collected from the vegetation in the woodland in the sable antelope breeding enclosures in the first year (July 2011–July 2012) than the second year (July 2012–July 2013) on the farm Hoopdal KQ96. The middle bar, the box and the whiskers refer to the median, the interquartile range and the interoctile (80%) range, whilst the dots are extremes or outliers.

Larvae of *A. hebraeum* were collected throughout the year in the multi-species enclosure, whilst none were collected in the sable enclosures during December 2011, April and August 2012, between October 2012 and May 2013 and in July 2013. Significantly more *A. hebraeum* larvae were collected in both the woodland (*V* = 198.0, *p* < 0.05, Figure 5) and the grassland habitats (*V* = 174.5, *p* < 0.01, Figure 6) in the multi-species enclosure than in the sable enclosures. There was no difference in the abundance of ticks recovered in woodland versus grassland habitats in the multi-species enclosure (*V* = 158.0, *p* = 0.83), or the sable enclosures (*V* = 59.0, *p* = 0.12). More *A. hebraeum* larvae ticks were collected in the first year (July 2011–July 2012) than in the second year (July 2012–July 2013) in the woodland habitat in the sable antelope enclosures (*V* = 28.0, *p* < 0.05, Figure 7).

#### Rhipicephalus appendiculatus

Significantly more *R. appendiculatus* larvae were collected in both woodland habitat (*V* = 231.0, *p* < 0.001) and grassland habitat (*V* = 195.5, *p* < 0.01) in the multi-species herbivore enclosure than in the sable antelope enclosures; and 91.1% (*n* = 2291) of all *R. appendiculatus* collected came from the multi-species enclosure. There was no significant difference in the total number of *R. appendiculatus* larvae collected in woodland versus grassland habitats in the multi-species enclosure (Wilcoxon signed rank test [paired by month] *V* = 157.5, *p* = 0.15), or the sable antelope breeding enclosures (*V* = 138.0, *p* = 0.44). *Rhipicephalus appendiculatus* accounted for 24.2% (*n* = 2515) of all ticks collected and was the only tick species of which nymphs (4.1% of all *R. appendiculatus* collected; *n* = 103) and adults were recovered frequently. Nymphs were collected in peak numbers from April to September of both years and none were recovered between October and March 2011/2012 or 2012/2013. The numbers of *R. appendiculatus* larvae collected in the multi-species enclosure had a distinct peak between March and May 2012, with a similar trend in 2013 and absolute peaks in April of both years. The numbers of *R. appendiculatus* larvae collected in the breeding enclosures were too low to significantly determine their seasonality.

#### Rhipicephalus decoloratus

*Rhipicephalus decoloratus* was collected throughout the year in both the multi-species enclosure as well as the sable breeding camps. It was the most abundant tick in the breeding enclosures, accounting for 65.4% (*n* = 2281) of all ticks collected in these enclosures, whilst it only accounted for 25.4% (*n* = 1676) of the ticks collected in the multi-species enclosure. Again, there was no significant difference between the abundance of *R. decoloratus* found in woodland versus grassland habitats for either the multi-species enclosure (*V* = 81.0, *p* = 0.09), or sable enclosures (*V* = 87.5, *p* = 0.21). However, in contrast to *R. appendiculatus* and *A. hebraeum* (where more ticks were found in the multi-species enclosure than in the sable enclosures), there was no significant difference between the numbers of *R. decoloratus* larvae collected in either woodland (*V* = 104.0, *p* = 0.31) or grassland (*V* = 1123.5, *p* = 0.66) in the multi-species enclosure or sable antelope enclosures.

**FIGURE 8:**
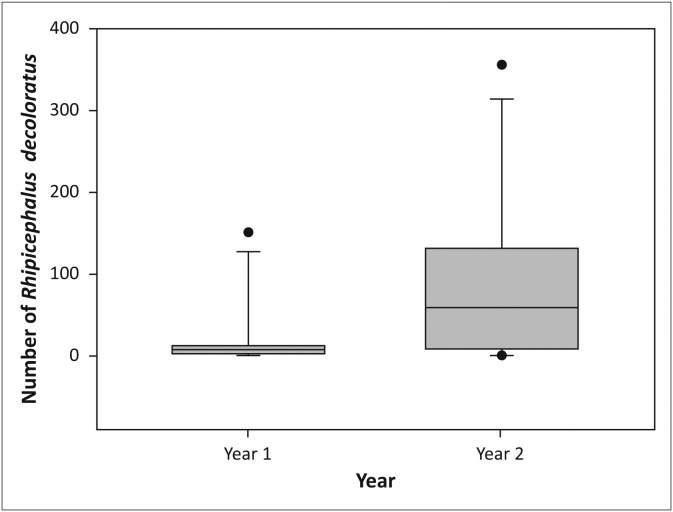
Boxplot depicting annual increase of *Rhipicephalus decoloratus* larvae collected from the vegetation in the grassland in the sable antelope breeding enclosures from the first year (July 2011–July 2012) to the second year (July 2012–July 2013) on the farm Hoopdal KQ96. The middle bar, the box and the whiskers refer to the median, the interquartile range and the interoctile (80%) range, whilst the dots are extremes or outliers.

*Rhipicephalus decoloratus* larvae were collected throughout the year, with peak collections in April and May 2012 and February and April 2013 in the multi-species enclosure, as well as in November 2012 and between October and December 2013 in the sable enclosures. There was a significant annual increase in the mean number of *R. decoloratus* larvae collected in the sable antelope breeding enclosures between July 2011 and July 2013 (*V* = 7.0, *p* < 0.05, Figure 8).

#### Rhipicephalus evertsi evertsi

**FIGURE 9:**
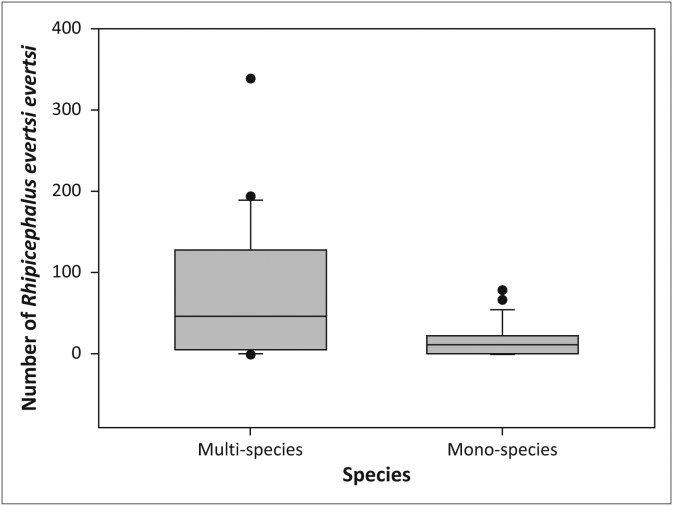
Boxplot depicting the number of *Rhipicephalus evertsi evertsi* larvae collected in grassland in the multi-species enclosure versus the sable antelope breeding enclosures on the farm Hoopdal KQ96. The middle bar, the box and the whiskers refer to the median, the interquartile range and the interoctile (80%) range, whilst the dots are extremes or outliers.

*Rhipicephalus evertsi evertsi* was the second most commonly collected tick species and accounted for 27.6% (*n* = 2866) of all ticks collected (Table 1). Although it was collected throughout the year in both the multi-species enclosure and the sable enclosures, there were distinct peaks in numbers between February and June 2012 and again between December 2012 and June 2013. More *R. evertsi evertsi* larvae were recovered in grassland than in woodland in the multi-species enclosure (*V* = 57.0, *p* < 0.01), but there was no difference in the abundance of ticks between habitats in the sable enclosures (*V* = 110.0, *p* = 0.86). There was no significant difference in the abundance of ticks in the woodland habitats (*V* = 149.0, *p* = 0.47) of the two sets of enclosures, but there were significantly more ticks collected in grassland in the multi-species enclosure than in the sable enclosures (*V* = 220.0, *p* < 0.05, Figure 9). Of the total number of *R. evertsi evertsi* larvae collected, 71.4% (*n* = 2047) were recovered in the multi-species enclosure (Table 1). This tick was the second most abundant species collected in the sable antelope breeding enclosures, where it accounted for 23.5% (*n* = 819) of the ticks collected (Table 1).

### Less commonly collected ticks

Four of the eight species of tick recovered were collected too infrequently to determine their relative or seasonal abundances. *Rhipicephalus zambeziensis* larvae were the most common of these and most were collected in woodland habitats between March and May 2013. Larvae and a single nymph of *R. simus* were collected and all collections were made between March and May of both 2012 and 2013. Two *A. marmorerum* larvae were collected in the woodland of the sable enclosures, one in July 2011 and the other in June 2012. Two adult female *H. elliptica* were collected in the multi-species enclosure in April 2012.

## Discussion

The ticks most commonly collected on the farm Hoopdal in this survey, namely *A. hebraeum, R. appendiculatus, R. decoloratus* and *R. evertsi evertsi*, were also the most commonly collected by Schroder *et al.* (2006) on the same farm*.*

*Rhipicephalus decoloratus* was significantly more abundant than *A. hebraeum* or *R. appendiculatus* in both woodland and grassland habitats in the sable antelope enclosures and accounted for 65.4% of the total number of ticks collected in the sable enclosures. Larvae were present throughout the year but the largest numbers were collected between October and December 2012. This resembles the seasonality of *R. decoloratus* collected at Skukuza in the Kruger National Park, where peak numbers of larvae were recovered in spring (September to November) and the lowest numbers in autumn and winter (Horak *et al.* 2011).

Most, larger wild ruminants, including impala, greater kudu and sable antelope, as well as Burchell’s zebra, have proved to be excellent hosts of *R.* *decoloratus*, a one-host species (Zieger *et al.* 1998b). The significantly greater abundance of *R. decoloratus* in both woodland and grassland habitats in the sable antelope enclosures when compared with the three-host ticks, *A. hebraeum* and *R. appendiculatus*, needs to be investigated further. It could result from a combination of the development of resistance to the synthetic pyrethroids used to treat the sables and their suitability as hosts for the tick as well as other factors, such as habitat suitability.

Resistance of ticks to synthetic pyrethroids is a serious worldwide problem and one which has been problematic for *R. decoloratus* in certain regions of South Africa (Mekonnen *et al.* 2003; Rodriguez-Vivas *et al.* 2011). The fact that questing and parasitic populations of *R. decoloratus* were seemingly unaffected by the regular application of synthetic pyrethroids to sympatric cattle on a wildlife/cattle ranch in Zambia has also been ascribed to the possible development of acaricide resistance (Zieger *et al.* 1998c).

Treatment of ranched wildlife with acaricides remains problematic and most current practices, such as the method described in this study, involve opportunistic and inaccurate treatment regimens. Accurate treatment of wildlife with an acaricide is only possible when the animal is immobilised and weighed. The current high value of ranched sable antelope in the commercial sector, coupled with the risks and costs associated with immobilisation, have resulted in ranchers seeking less invasive acaricide administration methods. Acaricide treatments that are not determined accurately according to body weight of the target species often result in the administration of non-lethal doses of acaricide (synthetic pyrethroids in this case) to the targeted tick species, which may in turn further accelerate the development of acaricide resistance. Some of the preferred attachment sites of *R. decoloratus* on cattle are the head, neck and shoulders. The method of acaricide application in the sable antelope enclosures, as described above, may, however, lead to the selection of highly resistant ticks, as they frequently come into direct contact with the acaricide-impregnated rolling pin, implying that those that survive this direct contact are likely to be highly resistant.

Although host suitability may also play a role in the significantly increased abundance of *R. decoloratus* in the sable breeding enclosures compared with *A. hebraeum* and *R. appendiculatus*, it is unlikely that it is a significant contributing factor. Plains zebra, which are excellent hosts of *R. decoloratus*, are one of the more abundant wildlife species in the multi-species enclosure, which they share with large numbers of impala and kudu, also excellent hosts of *R. decoloratus.* With the abundance of such a variety of excellent hosts as opposed to the single-host species in the sable antelope enclosures, it was to be expected that more *R. decoloratus* larvae would have been collected from the multi-species enclosure.

Despite good breeding results for roan and sable antelope in South Africa, confinement at high stocking rates may support the build-up of tick populations, as is the case with the population of *R. decoloratus* in this study. This may increase the challenge to naïve calves by ticks possibly carrying tick-borne diseases (Nijhof *et al.* 2005). The increasing incidence of fatal babesiosis in farmed sable may be related to the gradual over-abundance of *R. decoloratus* in intensive breeding enclosures, as recorded in this survey. Furthermore, animals in these enclosures are continuously exposed to a number of stress factors, both nutritional and social, as well as their own inability to select preferred habitat, thus increasing their susceptibility to disease.

Most of the preferred hosts of all stages of development of the three-host tick, *A. hebraeum*, are present on the farm Hoopdal, including giraffe, eland, kudu, impala, duiker and warthogs, whilst scrub hares (*Lepus saxatilis*), carnivores, ground-frequenting birds and leopard tortoises (*Stigmochelys pardalis*) are good hosts of the immature stages (Horak, Heyne & Donkin 2010; Horak *et al.* 1987, 2011). *Amblyomma hebraeum* larvae exhibited no significant seasonal abundance in this survey, but the slightly larger numbers collected in summer and lower numbers collected in July coincide with the observations made in similar surveys in other summer rainfall regions in South Africa (Horak *et al.* 2011). The largest numbers of *A. hebraeum* larvae were also collected during summer on Hoopdal by Schroder *et al.* (2006). Contrary to Horak *et al.* (2011), who collected significantly more *A. hebraeum* larvae in woodland than in grasslands in the Kruger National Park over a period of 164 months, there was no significant difference between the numbers of *A. hebraeum* larvae collected in woodlands and grasslands in this survey. The 2-year duration of the survey may have been too short to draw any inferences from this finding.

Significantly more larvae were collected in the multi-species enclosure than in the sable enclosures. Reasons for this difference could be the absence in the breeding enclosures of smaller mammals and ground-frequenting birds, which are important hosts for the immature stages of the tick (Horak *et al.* 1987), as well as the regular application of synthetic pyrethroids on the sable antelopes. In the Eastern Cape Province of South Africa, acaricidal treatment of sympatric cattle was considered to be a more significant factor in reducing the numbers of *A. hebraeum* on kudu than other factors such as the alteration of suitable habitat and availability of suitable hosts.

All stages of *R. appendiculatus*, a three-host tick, quest for their hosts from the vegetation (Spickett *et al.* 2011). These hosts include eland, kudu, waterbuck and smaller antelope, all of which are numerous on the farm Hoopdal. The peak in the numbers of *R. appendiculatus* larvae collected in April of both years during this survey was earlier than that recorded by Spickett *et al.* (2011) in the Kruger National Park, where larvae only peaked in June to August. The total numbers of *R. appendiculatus* larvae collected from woodland and grassland did not differ significantly in this survey. During a 164-month survey of ixodid ticks in the Kruger National Park, the habitat distribution of *R. appendiculatus* was variable in response to spatial use of the habitat by their preferred and other hosts (Spickett *et al.* 2011).

Only 8.91% of the 2515 *R. appendiculatus* larvae were collected in the intensive breeding camps. The low numbers collected from these camps can possibly be attributed to the regular tick control regimen applied to the sable antelopes. A similar reduction in numbers of *R. appendiculatus* on the vegetation, on impala on a commercial game ranch in Zambia and on kudu on a mixed cattle and game farm in the Eastern Cape Province, South Africa, on which intensive tick control was practised on sympatric cattle, has been observed (Horak & Knight 1986).

*Rhipicephalus evertsi evertsi* was the second most abundant tick collected overall in this survey, as well as being the second most abundant species collected in the sable antelope breeding enclosures. Burchell’s zebra, greater kudu and impala, as well as a few eland, all of which are preferred hosts of all stages of development of this two-host tick (Zieger *et al.* 1998b), are present on the property. The relative abundance of *R. evertsi evertsi* (23.47%) in the intensive breeding camps could also be attributed to the development of acaricidal resistance. The immature stages of this two-host tick remain on the host for longer than those of three-host ticks and more than one life cycle can be completed annually, thus allowing more exposure over time to the acaricides with resultant perpetuation of the resistant genes in the population.

Only two *A. marmoreum* larvae were collected in this study, one in July 2011 and one in July 2012. The preferred host of the adults of this tick is the leopard tortoise (*S. pardalis*), which implies that one or more tortoises either were or had been present in the breeding enclosures (Horak *et al.* 2006).

Two adult *Haemaophysalis elliptica* were collected in this study. The adults of this tick have frequently been recovered from a number of large and small carnivore species, including leopards (*Panthera pardus*) and black-backed jackals (*Canis mesomelas*), both of which occur erratically on the farm Hoopdal. Five of the seven *R. simus* collected in this study were collected in woodland habitats and two of the seven ticks recovered were collected in the multi-species herbivore enclosure. The preferred hosts of adult *R. simus* include Burchell’s zebra and warthogs, whilst those of the immature stages are rodents and scrub hares, which are all abundant on the farm Hoopdal (Horak, Fourie & Braack 2005; Walker *et al.* 2000).

Small numbers of *R. zambeziensis* larvae have been collected from the vegetation in the Thabazimbi district as well as from that on the farm Hoopdal (Schroder *et al.* 2006; Uys & Horak 2005), a region where the distribution of *R. appendiculatus* and *R. zambeziensis* are considered to overlap. In the present survey, 14.7% of all *R. zambeziensis* larvae were collected in April and May 2012, whilst 67.2% were collected between March and May 2013.

## Conclusion

The commercial game farming practice of breeding rare and endangered antelopes such as sable antelope in mono-species enclosures where strategic tick control is practised may have a significant impact on the population dynamics of the one-host tick, *R. decoloratus*, which possibly increases in abundance over time within these breeding enclosures*.* Observations made on other tick species, such as *A. hebraeum* and *R. evertsi evertsi*, suggest that this practice may also affect their population dynamics and this should be investigated further. The progressive development of over-abundance of any tick species that is potentially a vector of fatal tick-borne diseases such as babesiosis and theileriosis, despite strategic control, is of great concern. Recent studies on the evolution of acaricidal resistance in *Rhipicephalus microplus* suggest that alternative strategies to reduce acaricidal resistance, other than the rotation of synthetic pyrethroids, which is ineffective, should be sought (Rodriguez-Vivas *et al.* 2011). The breeding of wildlife species, such as sable antelope, in isolated enclosures where tick control is practised is unlikely to prevent the loss of animals to fatal tick-borne diseases in the long term. A reason for this is that the population dynamics of the tick vectors, which potentially transmit these diseases, are also affected, leading to their over-abundance, which in turn may lead to an increased challenge of tick-borne diseases such as babesiosis and theileriosis. When compared with the incidence of disease, the population dynamics of ticks in intensive breeding enclosures may give important insights into their potential vector status for tick-borne diseases in sable antelope. Alternative methods of tick control should also be sought, including rotational grazing systems and the correct wildlife species composition in enclosures.
